# The Complete Genome Sequence of a Human Parechovirus from a Child with Diarrhea in China Revealed Intertypic Recombination

**DOI:** 10.1128/genomeA.00332-17

**Published:** 2017-05-25

**Authors:** Xiaoying Zhao, Chenglin Zhou, Xiaodan Zhang, Wang Li, Xinyu Wan, Yan Wang, Yuming Zeng, Wen Zhang

**Affiliations:** a Department of Microbiology, School of Medicine, Jiangsu University, Zhenjiang, Jiangsu, China; b Department of Laboratory Medicine, Jiangsu Taizhou People’s Hospital, Taizhou, Jiangsu, China; c The Zhenjiang Center for Disease Control and Prevention, Zhenjiang, Jiangsu, China

## Abstract

A human parechovirus (HPeV), CH-ZXY1, was detected in feces from a child with diarrhea. Phylogenetic trees over three different genomic regions revealed discordant topological structures. Recombination analysis indicates that CH-ZXY1 is a recombinant resulting from recombination between HPeV5 and HPeV1, which was confirmed by PCR covering the recombination breakpoint.

## GENOME ANNOUNCEMENT

Human parechovirus (HPeV) belongs to the *Parechovirus* genus of the *Picornaviridae* family, which include nonenveloped, positive-sense RNA viruses with icosahedral capsids (1–3). The genome of HPeV is about 7,300 nucleotides (nt) in length encoding a single large open reading frame (ORF), which comprised three regions P1, P2, and P3 and encodes a polyprotein posttranslationally cleaved into three structural proteins and seven nonstructural proteins. HPeVs were shown to be highly diverse, with up to 16 provisionally assigned types (4, 5), where HPeV1 and HPeV2 were originally known as echoviruses 22 and 23 (6). Most of the HPeV infections are mild, including diarrhea and respiratory tract infection (7, 8).

From January to December 2014, a total of 100 fecal samples were collected from children < 6 years of age with acute diarrhea who were treated as outpatients or hospitalized at the Affiliated Hospital of Jiangsu University. The 100 fecal samples were prepared into 10 sample pools and subjected to viral metagenomic analysis (9, 10). One library contained 18,823 sequence reads showing sequence similarity to HPeVs, which could be assembled into a nearly complete genome. The assembled genome was then confirmed by PCR with four sets of primers.

The nearly complete genome, named CH-ZXY1, consists of 7,213 nt and includes an ORF beginning at nt 613 and ending at nt 7,173, encoding a putative polyprotein precursor of 2,187 amino acids. A BLASTn search in GenBank showed that the compete genome of CH-ZXY1 shared the highest sequence similarity of 84% to an HPeV5 strain (JX050181), and 76% to 84% to the other HPeV genomes available in GenBank.

Based on P1 and P2 gene regions, CH-ZXY1 phylogenetically clustered with the other HPeV5 strains, while in the P3 gene region, CH-ZXY1 was closely related to HPeV1 strains, sharing the highest sequence identity of 91% with an HPeV1 strain (KC769584), suggesting recombination might occur here. Recombination analysis was performed over the complete genomes of CH-ZXY1 and related strains with RDP4.0 software. One potential recombination event was found which occurred between the lineage of HPeV5 represented by the strain Br/53/2006 (HQ696575) and the lineage of HPeV1 represented by the strain BNI-788st (EF051629). Br/53/2006 was identified in fecal samples from patients with acute diarrhea in Brazil (11), while BNI-788st was isolated from the stool sample of a German patient in Hamburg (12). Bootscan analysis showed one potential breakpoint located at the border between the P2 and P3 regions. Further PCR amplification with primers covering the potential recombination breakpoint was performed to confirm the recombination event. The PCR products were sequenced and showed identical to the original sequence.

Homologous genetic recombination plays an important role in the evolution of almost all genera of picornaviruses (13–17). Intratypic recombination among HPeVs more frequently observed (18–20) though intertypic recombination was also reported (4, 21). This study reported the first intertypic recombination occurring between HPeV5 and HPeV1 leading to an HPeV5 recombinant, which may highlight the evolutionary dynamics and diversity of HPeVs.

### Accession number(s).

This genome sequence of CH-ZXY1 has been deposited in GenBank under the accession no. KY067444.
